# Brief Azacytidine Step Allows The Conversion of Suspension Human Fibroblasts into Neural Progenitor-Like Cells 

**DOI:** 10.22074/cellj.2015.522

**Published:** 2015-04-08

**Authors:** Fahimeh Mirakhori, Bahman Zeynali, Sahar Kiani, Hossein Baharvand

**Affiliations:** 1 School of Biology, College of Science, University of Tehran, Tehran, Iran; 2 Department of Stem Cells and Developmental Biology at Cell Science Research Center, Royan Institute for Stem Cell Biology and Technology, ACECR, Tehran, Iran; 3 Department of Developmental Biology, University of Science and Culture, ACECR, Tehran, Iran

**Keywords:** Azacytidine, Fibroblast, Progenitor Cells, Transdifferentiation

## Abstract

In recent years transdifferentiation technology has enabled direct conversion of human fibroblasts to become a valuable, abundant and accessible cell source for patient-specific induced cell generation in biomedical research. The majority of transdifferentiation approaches rely upon viral gene delivery which due to random integration with the host genome can cause genome instability and tumorigenesis upon transplantation. Here, we provide a simple way to induce neural progenitor-like cells from human fibroblasts without genetic manipulation by changing physicochemical culture properties from monolayer culture into a suspension in the presence of a chemical DNA methyltransferase inhibitor agent, Azacytidine. We have demonstrated the expression of neural progenitor-like markers, morphology and the ability to spontaneously differentiate into neural-like cells. This approach is simple, inexpensive, lacks genetic manipulation and could be a foundation for future chemical neural transdifferentiation and a safe induction of neural progenitor cells from human fibroblasts for clinical applications.

Fibroblasts are found in the majority of animal tissues, particularly in our largest organ, the skin. Their presence in the skin provides an abundant supply of fibroblasts which are easy to obtain by a simple biopsy. In recent years, with the advent of transdifferentiation technology, they have gained additional attention as a valuable cell source for patient-specific induced cell generation (1, 2). Differentiated somatic cells can imbue new differentiation potential into desired cells such as neural progenitor cells (NPCs) through transgenic approaches (2-4). However, the majority of these approaches rely upon viral gene delivery which due to random integration with the host genome can cause genome instability, insertional mutations and tumorigenesis upon transplantation which is of concern (2). Although non-integrating approaches such as plasmids, or RNA and proteins of desired keystone genes have been developed to lessen these risks, however their low efficiency remains an issue (2, 5, 6). Notably, many recent studies have reported that the specific culture environment such as low O_2_ concentrations and the presence of epigenetic chemical modifiers can simply trigger cellular transdifferentiation (7-12). They showed that the culture environment such as the hypoxic condition in the form of 5% O_2_ or sphere environment can initiate reprogramming of mouse fibroblasts into multipotency. Such findings suggest a pivotal role of the culture environment in reprogramming and transdifferentiation, and may offer development toward genetic material free induction approaches for a patient’s own cell-derived cells of choice.

Here, we changed the human foreskin fibroblasts conventional culture condition from a monolayer to a suspension in the presence of Azacytidine (Aza; an epigenetic chemical DNA methyltransferase inhibitor) with the intent to investigate whether neural progenitor like-cells could be induced in these cells without any genetic manipulation.

As reported by Su et al. (11), forcing cells to undergo cell-cell contact instead of cell-matrix in sphere-like structures could lead to neural progenitor related gene expression in mouse embryonic fibroblasts and induced neural progenitor production. Thus, we initially attempted to test the ability of human foreskin fibroblasts (HFFs) to form sphere-like structures under suspension culture. We cultured HFF on agarose-coated plates and found that in the suspension culture they formed sphere-like aggregates which were less than 100 μM in diameter after 18-24 hours (Fig.1A). Quantitive polymerase chain reaction (Q-PCR) demonstrated that under this condition HFFs expressed neural progenitor gene markers *SOX2* and *PAX6*. In addition, there was significant upregulation of *NESTIN* when compared with cells cultured under monolayer conditions. The fibroblast specific protein 1 (*FSP1*) downregulated in these cells (Fig.1B). As these cells expressed NPC-related markers, we examined whether they had the potential to proliferate toward a neural progenitor-like lineage. Thus, we cultured these cells on polyornithine/laminin-fibronectin (PLF) coated plates. Despite gene expression, further culturing on PLF-coated plates for another two weeks showed no immunoreactivity for NPC markers such as *NESTIN, PAX6* or *SOX2* and no apparent morphological changes (data not shown).

Several reports thus far have demonstrated that mouse fibroblasts can convert to NPCs and multipotent stem cells by a suspension culture (7, 11). However, these results showed that HFF formed sphere-like structures that expressed NPC markers under a suspension culture, but unlike mouse fibroblasts they could not simply convert into neural progenitor-like cells.

The formation of spheres alone could not account for increased induction of NPC traits in HFFs. Therefore we tested the implementation of a brief Aza treatment according to the protocol of Pennarrosa with modifications (13), as outlined in figure 2A. Cells were cultured in suspension and treated overnight with 1 μM Aza after which Aza was removed from the culture. In the monolayer culture after 2 days of Aza treatment, we observed detached, nonviable cells. Interestingly, cells treated under suspension culture formed smaller aggregates compared to the untreated spheres (~30-50 μM diameter sized spheres) and survived for several days. Upon cultivation for 14 days under this inductive condition, the expressions of *SOX2, NESTIN* and *PAX6* upregulated and FSP1 was downregulated. In addition, the treated cells expressed higher levels of other neural progenitor markers (*EN1, LMX1A* and *WNT1*). Surprisingly, there was a striking difference in *SOX2, PAX6*, and *EN1* expression in the Aza-treated group compared to the untreated cells (Fig.2B). Next, we transferred single cells onto PLF-coated plates for an additional two weeks and observed that these cells became NPC-like in morphology. Cells became smaller, acquired radial arrangement and produced neurosphere-like aggregates from adherent culture spontaneously which were passagable (Fig.2C). Immunocytochemical analysis demonstrated that these cells were positive for *NESTIN, PAX6*, and *SOX2* (Fig.2D, Table 1). Subsequently we tested whether the resultant cells could be differentiated into neural cells. Our results showed that following withdrawal of growth factor for two weeks, these cells expressed a neuronal marker TUJ1 and the astrocytic marker GFAP (Fig.2D). The oligodendrocyte marker O4 was not observed (data not shown). These results indicated the presence of another NPC-like property in these cells-the ability to differentiate into neurons and astrocytes *in vitro*.

Aza has been previously reported to improve reprogramming and transdifferentiation of HFF toward pancreatic progenitors (13). However, its effect on neural progenitor induction is largely unknown. In the present study, for the first time, we have reported that this protocol gradually induced a neural program in HFF and cells that resembled NPC morphology emerged after 28 days. These cells were positive for NPC-related markers and could differentiate into neuronal cells. The expressions of PAX6 and mid-brain neural progenitor markers such as EN1, LMX1A, and WNT1 suggested a possible bias toward a more specific neural fate.

Here, we introduced a reliable, simple protocol that induced NPC-like properties into HFF by the change in conventional culture conditions. This protocol may open a new platform for the possible chemical approach to generate NPCs from a patient’s own fibroblasts, which would eliminate the use of genetic manipulation. However, additional studies such as functional tests and analyses, the purity of the cells expressing neural progenitor-like markers and also other chemical agents enhancing the efficiency required to prove this hypothesis and may be helpful to get a very rough idea of how efficient such a new method could be.

**Fig.1:**
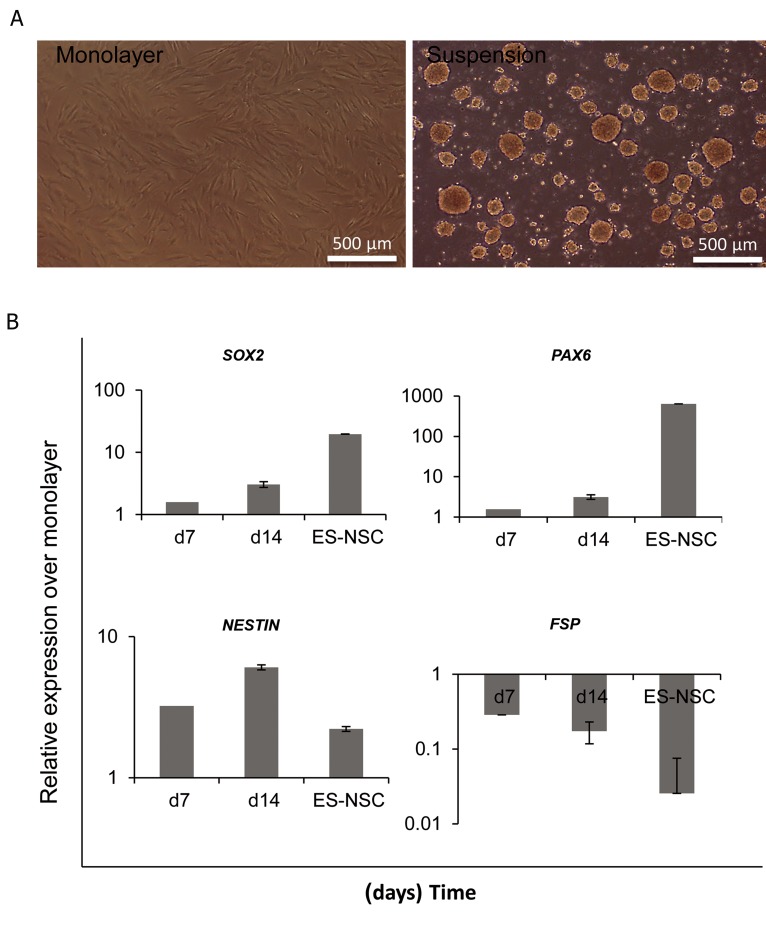
Induction of neural progenitor related genes in HFFs. A. Morphology of human fibroblasts in monolayer and suspension culture and B. Q-PCR analysis of cells under suspension culture for NSC markers. HFFs; Human foreskin fibroblasts, Q-PCR; Quantitive-polymerase chain reaction and ES-NSC; Embryonic stem cell-drived neural stem cell.

**Fig.2:**
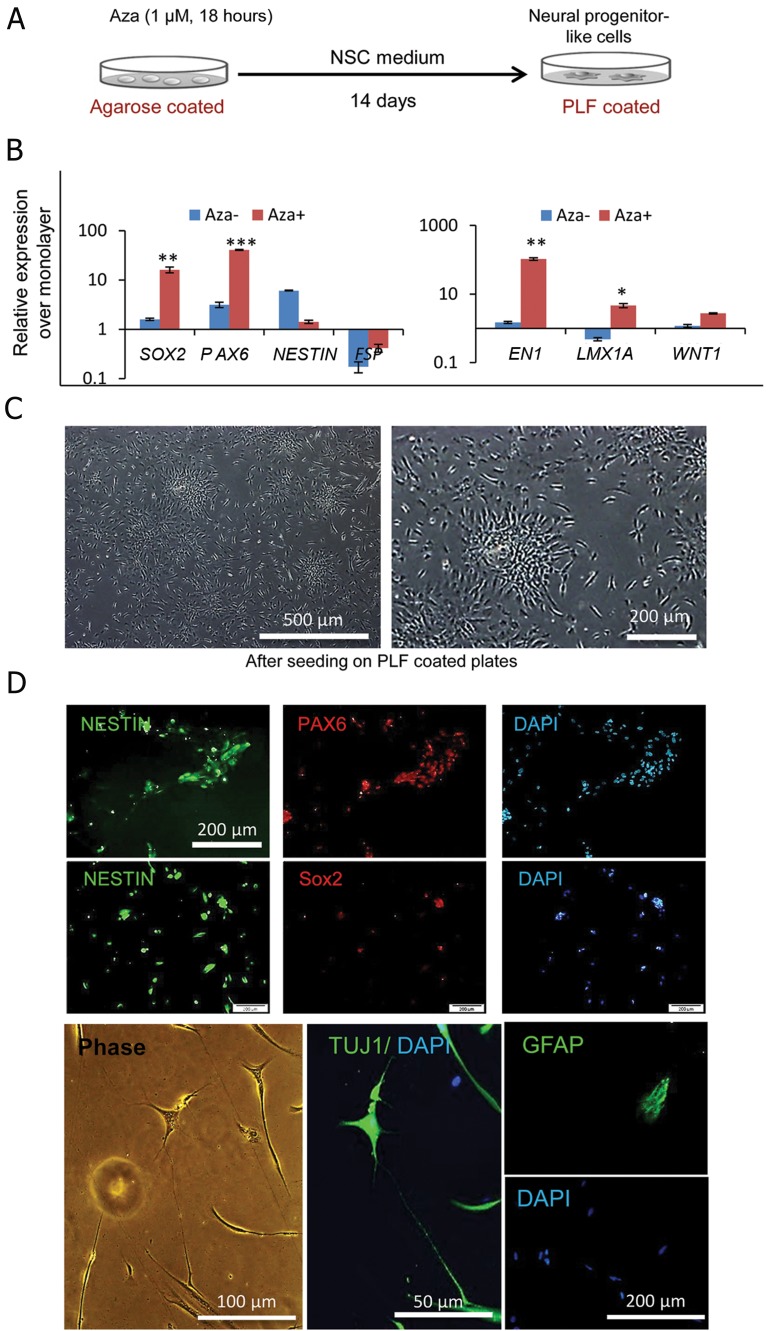
Induction of neural progenitor like traits in HFFs via azacytidine (Aza) treatment. A. Schematic design of the induction protocol, B. Q-PCR for NSC related genes in Aza treated and untreated HFFs under suspension culture, C. The morphological changes of Aza treated HFFs after 2 weeks on PLF coated plates and D. Immunocytochemistry of the treated cells showed positive immunoreaction for neural progenitor markers Nanog, SOX2 and PAX6 after 4 weeks in culture. After growth factor withdrawal, a few cells were positive for TUJ1 and GFAP. HFFs; Human foreskin fibroblasts, NSC; Neural stem cell and PLF; Polyornithine/laminin-fibronectin.

**Table 1:** Primer name and sequences were used in this study


Gene name	Primer sequences

**SOX2**	F: 5´GGAGTGCAATAGGGCGGAAT3´
	R: 5´CCA GTT GTA GAC ACG CAC CT3´
**PAX6**	F: 5´GTC CAT CTT TGC TTG GGA AA3´
	R: 5´TAG CCAGGT TGCGAA GAA CT3´
**NESTIN**	F: 5´CTC CAG AAA CTC AAG CAC C3´
	R: 5´TCC TGA TTC TCC TCT TCC A3´
**GAPDH**	F: 5´CTC ATT TCC TGG TAT GAC AAC GA 3´
	R: 5´CTT CCT CTT CTC CTC TTG CT 3´
**FSP1**	F: 5´ACT TGG ACA GCA ACA GGG AC3´
	R: 5´CCC CAA CCA CAT CAG AGG AG3´
**EN1**	F: 5´CGCAGCAGCCTCTCGTATGG3´
	R: 5´GCCGCTTGTCCTCCTTCTTCG3´
**LMX1A**	F: 5´GCCTCATTTGAAGTATCCTCC3´
	R: GCTTCTTCATCTTCGCTCTC3´
**WNT1**	F: 5´CCTCCACGAACCTGCTTACA3´
	R: 5´TCGGGTGACGATCTTGCCGAA3´

